# The Salinogenic Food Environment: A Conceptual Framework Linking Salt Exposure, Dietary Behaviors, and Cardiovascular Health

**DOI:** 10.3390/nu18203454

**Published:** 2026-10-21

**Authors:** Carla Gonçalves, Tânia Silva-Santos, Pedro Moreira

**Affiliations:** 1 Department of Biology and Environment, School of Life Sciences and Environment, University of Trás-os-Montes and Alto Douro, 5000-801 Vila Real, Portugal; taniasantos@utad.pt; 2 EPIUnit ITR, Institute of Public Health of the University of Porto, Rua das Taipas, No. 135, 4050-600 Porto, Portugal; pedromoreira@fcna.up.pt; 3 RISE-Health, School of Life and Environmental Sciences, University of Trás-os-Montes and Alto Douro (UTAD), 5000-801 Vila Real, Portugal; 4 CITAB-Centre for the Research and Technology of Agro-Environmental and Biological Sciences, University of Trás-os-Montes and Alto Douro, 5000-801 Vila Real, Portugal; 5 Faculty of Nutrition and Food Sciences, University of Porto, 4099-002 Porto, Portugal

**Keywords:** cardiovascular health, dietary behavior, food environment, food policy, public health, salt reduction

## Abstract

**Background**: Excessive sodium intake is a major modifiable risk factor for hypertension and cardiovascular disease and remains well above recommended levels globally. Although sodium reduction strategies have traditionally focused on individual behaviour change, food environments largely determine sodium consumption by shaping food availability, affordability, preferences, and purchasing options. However, no conceptual framework has specifically characterized food environments according to their capacity to promote excessive salt intake. **Objectives**: To introduces the concept of the salinogenic food environment and proposes a conceptual framework integrating environmental determinants of sodium exposure across food systems, dietary behaviors and cardiovascular health outcomes. **Methods**: A narrative conceptual review synthesized evidence from ecological health models, particularly the ANGELO framework, food environment research, sodium epidemiology, dietary behaviours, and cardiovascular health. Evidence from retail, out-of-home and institutional food settings, sociocultural and economic influences, and policy environments informed framework development. **Results**: The salinogenic food environment is defined as an environment that promotes high salt intake through the widespread availability of high-sodium foods and the reinforcement of salty taste preferences. The framework encompasses four interrelated domains: physical, economic, sociocultural, and policy/governance environments. Evidence indicates that processed and ultra-processed foods, restaurant and institutional meals, food pricing structures, cultural dietary practices, and insufficient regulatory measures collectively contribute to excessive sodium exposure. The framework highlights how these domains interact to shape sodium exposure, reinforce salty taste preferences, and contribute to socioeconomic inequalities in sodium intake. It also proposes approaches for operationalizing salinogenic environments through objective indicators, perceived environment measures, and composite indices. **Conclusions**: The salinogenic food environment framework reorients sodium reduction from an individual responsibility toward to a structural property of contemporary food systems. By providing a common conceptual language for research, surveillance and policy evaluation, it may facilitate the development of more effective multisectorial strategies to reduce sodium exposure and improve cardiovascular health.

## 1. Introduction

Non-communicable diseases (NCDs) are the leading cause of death globally, accounting for approximately 71% of all mortality [1]. These conditions, which include cardiovascular diseases, diabetes, and certain cancers, are intricately linked to lifestyle factors, rapid urbanization, and societal changes [2]. To understand these links, ecological models conceptualize these health outcomes as the result of interactions—a “settling point”—between biological, behavioral, and environmental influences [3,4]. A widely adopted tool for dissecting these influences is the ANGELO framework (Analysis Grid for Environments Linked to Obesity/Health), which classifies environments according to size (micro vs. macro) and type (physical, economic, political, and sociocultural) [3]. Within these frameworks, environmental determinants are recognized as primary drivers of dietary patterns, as individuals often struggle to make healthy choices in surroundings that aggressively promote the consumption of energy-dense and high-salt foods [3].

Excessive sodium consumption is a well-established cause of raised blood pressure, which is the leading modifiable risk factor for cardiovascular disease (CVD) [5]. CVDs are responsible for approximately 32% of all deaths worldwide [6]. In response, the World Health Organization (WHO) has established a strong recommendation for adults to consume less than 2 g of sodium (equivalent to 5 g of salt) per day [7]. Despite these recommendations, the global mean salt consumption is estimated to be approximately 10 g per day, double the recommended limit [8]. This excessive sodium intake is responsible for an estimated 1.71 to 1.89 million deaths annually and 37 to 41 million disability-adjusted life years (DALYs) globally [9]. Consequently, WHO member states have committed to a global target of a 30% relative reduction in population salt intake by 2025/2030 [1,10].

While the concept of the “obesogenic environment” has been extensively utilized to characterize settings that promote weight gain through energy imbalance [3,11], no equivalent framework exists to characterize environments according to their capacity to promote excessive salt consumption. Most public health strategies have historically focused on individual-level behavioral changes and education, which have met with limited success because they fail to address the “upstream” drivers of intake [3,12], consistent with the Ottawa Charter’s emphasis on creating supportive environments for health [13]. Existing food environment frameworks successfully characterize determinants of overall diet quality but do not explicitly identify the environmental characteristics that drive sodium overexposure [12]. There is, therefore, an urgent need for a conceptual expansion that explicitly addresses the unique domains of the salt-promoting environment. Rather than replacing existing food environment models, the proposed framework complements them by explicitly identifying the environment features that promote excessive sodium exposure. The present review addresses this gap by introducing the concept of the salinogenic food environment and providing an integrated structure for its characterization, measurement, and application to research and public health policy.

The aim of this study is to propose and conceptualize the concept of a salinogenic food environment, defined as an environment that promotes high salt intake through the availability of high-sodium foods and the fostering of salty taste preferences [14]. By aligning this concept with established food environment models like the ANGELO framework [3], this paper seeks to provide a comprehensive structure for identifying the physical, economic, political, and sociocultural drivers of salt consumption. Ultimately, this framework intends to shift the public health focus from individual responsibility toward structural and environmental interventions, providing a framework to identify priorities for policy action and food system transformation.

## 2. Literature Search and Selection

The literature search was planned to identify relevant evidence across the multiple dimensions that potentially contribute to a salinogenic food environment. Relevant literature was identified through searches in PubMed, Scopus, and Web of Science, supplemented by targeted searches in reference lists and reports from national and international public health organizations available up to April 2026.

The search terms were used in different combinations and included terms related to sodium and salt exposure (“salt”, “sodium”, “dietary sodium”, “salt intake”, and “salt reduction”) along with terms addressing food environments and their main dimensions (“food environment”, “food system”, “obesogenic environment”, “ANGELO framework”, “processed foods”, “ultra-processed foods”, “eating out”, “financial accessibility to food”, “socioeconomic inequalities”, “preference for salty taste”, “sociocultural factors”, “food policy”, “food reformulation”, “public food procurement”, and “food labeling”). Additional targeted research addressed the assessment of sodium intake, hypertension, and cardiovascular health.

Given the narrative purpose of the review, reference studies and reports were selected based on their relevance to the conceptual development and characterization of the proposed framework.

Publications were excluded when they did not address dietary sodium/salt exposure or were not relevant to the environmental-to-health pathway considered in this review. Duplicate publications and sources that did not contribute additional conceptual or empirical information were also excluded.

The evidence-selection workflow involved four stages: (1) broad identification of potentially relevant literature through database and targeted searches; (2) assessment of titles and abstracts for conceptual relevance; (3) examination of full texts and authoritative reports for their contribution to the proposed framework; and (4) thematic allocation of the selected evidence to the physical, economic, sociocultural, policy/governance, measurement, behavioral, or health-outcome components of the framework. Seminal conceptual publications were retained where necessary to establish key constructs, whereas recent systematic reviews, authoritative guidelines, population-level studies, and studies using objective sodium-exposure measures were prioritized when characterizing.

Generative Artificial Intelligence was used to assist with the preparation of graphical elements included in the figures. All AI-assisted outputs were critically reviewed and edited by the authors, who take full responsibility for the final content of the figures.

## 3. Conceptual Framework: The Salinogenic Food Environment

### 3.1. Definition

A salinogenic food environment is formally defined as an environment that promotes high salt intake, either through the ubiquitous availability of foods with high sodium content or by fostering dietary habits that lead to the development of a taste preference for salty flavors [14]. This definition aligns with ecological health models, such as the ANGELO framework [3], which views health outcomes as the “settling point” of complex interactions between biological factors and external environmental influences. In a salinogenic context, excessive consumption is not merely an individual choice but an adaptive response to an environment where salt is used extensively by the food industry and catering sectors [15]. Accordingly, the concept encompasses both immediate environment exposure to high-sodium foods and the longer-term shaping of salty taste preferences.

### 3.2. Domains

**Figure 1:**
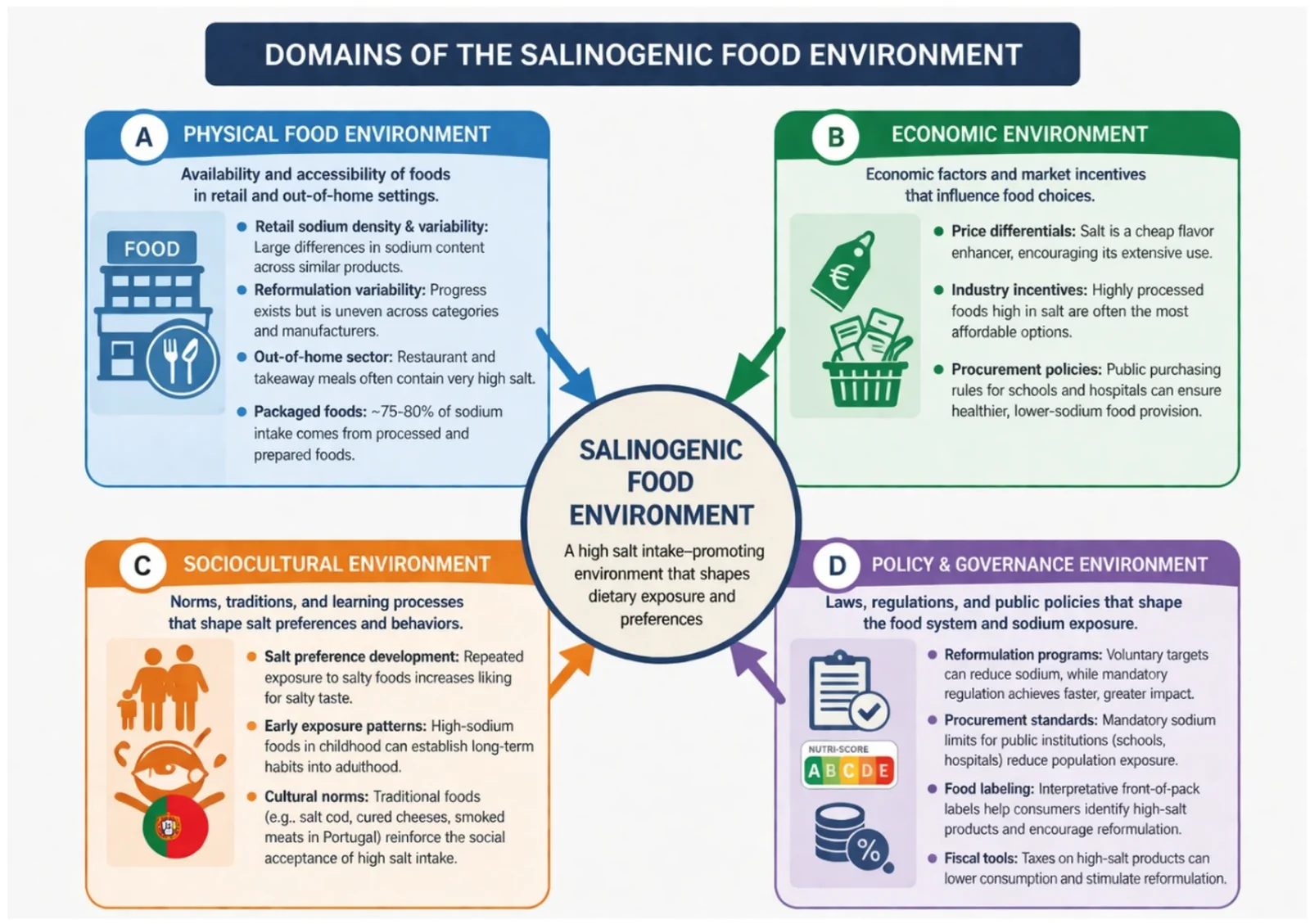
Domains of the salinogenic food environment.

The salinogenic food environment can be conceptualized as a multidimensional system encompassing physical, economic, sociocultural, and policy-related domains. These domains interact to shape population-level exposure to dietary sodium and influence consumption patterns across different contexts (Figure 1).

#### 3.2.1. Physical Food Environment

The physical food environment refers to the availability and accessibility of foods within retail settings and the out-of-home sector and represents a primary determinant of sodium exposure. In retail environments, there is considerable variability in sodium content across similar products, indicating that high sodium levels are often a consequence of formulation practices rather than technological necessity. Although reformulation initiatives have achieved reductions in certain food categories, such as bread and breakfast cereals, progress remains inconsistent across regions and manufacturers [16].

The out-of-home sector further amplifies sodium exposure. Meals consumed in restaurants, canteens, and takeaway outlets frequently contain excessive amounts of salt, with some single portions exceeding the WHO recommended daily intake of 5 g [12]. This is particularly relevant given the increasing reliance on food prepared outside the home. Overall, in high-income countries, approximately 75-80% of dietary sodium intake originates from processed foods and prepared meals, underscoring the central role of the physical food environment in shaping dietary exposure [15].

#### 3.2.2. Economic Environment

The economic environment influences dietary behavior through pricing structures, market incentives, and resource distribution. Salt is a low-cost ingredient widely used by the food industry and catering sector to enhance palatability, which incentivizes its extensive use in processed and prepared foods [5]. Consequently, products with high sodium content are often more affordable and widely available than healthier alternatives.

Highly processed and ultra-processed foods, frequently rich in sodium to improve shelf life and mask undesirable flavors, tend to dominate lower-cost food options [15]. This creates structural barriers to healthier dietary choices, particularly among socioeconomically disadvantaged populations. In this context, public procurement policies represent a critical economic lever. Regulating the nutritional quality of foods provided in institutional settings, such as schools and hospitals, can substantially influence both supply and consumption patterns, contributing to reduced population-level sodium intake [15,17].

#### 3.2.3. Sociocultural Environment

The sociocultural environment encompasses the norms, traditions, and learned behaviors that shape dietary practices and preferences. Repeated exposure to high-sodium foods can alter taste perception, leading to habituation and a preference for increasingly salty flavors [18]. This process is particularly influential during early life, when taste preferences are formed and reinforced. Exposure to high-sodium foods during childhood, such as through school meals or family dietary patterns, can establish long-term consumption habits that persist into adulthood.

Cultural norms and culinary traditions also play a significant role in maintaining high salt intake. In many contexts, traditional foods with elevated sodium content are deeply embedded in cultural identity and social practices. For example, in Portugal, foods such as salted cod, cured cheeses, olives, and processed meats are integral to national gastronomy, reinforcing the social acceptability of a high-salt diet [14]. These sociocultural factors can present important challenges to population-level salt reduction strategies.

#### 3.2.4. Policy & Governance Environment

The policy and governance environment include the regulatory frameworks, institutional policies, and public health strategies that shape the food system. Reformulation programs have been widely implemented as a key strategy to reduce sodium levels in processed foods. Evidence from countries such as the United Kingdom demonstrates that voluntary reformulation targets, when supported by monitoring and accountability mechanisms, can contribute to meaningful reductions in population sodium intake. However, mandatory regulatory approaches are generally more effective in achieving faster and more consistent outcomes.

Public procurement standards represent another critical policy tool. The implementation of mandatory sodium limits for foods served in public institutions, including schools, hospitals, and government facilities, can significantly reduce exposure among large population groups. In addition, interpretive front-of-pack labeling systems (such as traffic light labels or warning symbols) facilitate consumer understanding of nutritional content, help identify “hidden” sources of salt, and incentivize product reformulation by manufacturers.

Fiscal measures have also emerged as effective interventions. Taxes on high-salt foods, as implemented in countries such as Hungary, have been associated with reductions in the consumption of salty products and have encouraged industry reformulation [19]. Together, these policy approaches highlight the importance of a comprehensive, multisectoral strategy to address the salinogenic nature of contemporary food environments.

## 4. Measurement of Salinogenic Environments

The operationalization of the salinogenic food environment requires a comprehensive measurement approach that integrates objective indicators of sodium exposure with measures of perception and behavior. Such an approach enables the quantification of both the structural determinants of sodium availability and the adaptive responses of individuals within these environments. Accordingly, measurement should focus not only on sodium exposure but also on the environmental characteristics that generate and sustain it, including food availability, affordability, accessibility, marketing, and institutional practices.

### 4.1. Objective Indicators

Objective indicators are essential for quantifying the extent to which food environments promote sodium exposure across different settings.

Retail environments represent a primary source of dietary sodium, particularly in high-income countries, where approximately 75–80% of intake is derived from processed and packaged foods. Measurement of sodium density within retail settings reveals substantial variability across products within the same category, indicating considerable scope for reformulation. For example, studies have shown wide ranges in sodium content in staple products such as bread, with higher-sodium options contributing disproportionately to daily intake targets compared with lower-sodium alternatives. Monitoring systems, including platforms such as FoodSwitch and nutrient composition databases (e.g., EuroFIR), provide valuable tools for systematically tracking sodium levels across food supplies and identifying reformulation benchmarks [15].

The out-of-home food environment is another critical contributor to salinogenic exposure. Meals consumed in restaurants, takeaway outlets, and institutional catering settings frequently contain excessive amounts of salt, in some cases exceeding the WHO recommended daily limit of 5 g in a single serving. Menu-based sodium audits have consistently demonstrated high sodium densities in commonly consumed items, such as pizzas and Asian-style takeaway meals. These objective assessments are particularly important given that consumers tend to underestimate the sodium content of foods consumed outside the home.

Institutional food environments, including schools, hospitals, and workplaces, provide important opportunities for controlled interventions. Objective indicators in these settings include the implementation and compliance with mandatory nutritional standards for food provision. Evidence from school-based interventions, such as national programs targeting menu reformulation and food handler training, demonstrates that significant reductions in sodium content can be achieved at scale, particularly through modifications to commonly consumed items such as soups and staple dishes.

While environmental indicators quantify sodium availability, 24 h urinary sodium excretion remains the gold standard for assessing actual population exposure. This biomarker captures total sodium intake from all sources, including both processed foods and discretionary salt added during cooking or at the table, thereby overcoming limitations associated with self-reported dietary assessment methods.

### 4.2. Perceived Environment

In addition to structural exposure, the salinogenic environment operates through perceptual and behavioral mechanisms that shape dietary preferences and choices.

A central component is the adaptation of taste preferences. Repeated exposure to high-sodium foods leads to a progressive increase in the preferred level of saltiness, reinforcing habitual consumption patterns. However, evidence indicates that salt taste perception is highly adaptable. Gradual reductions in sodium content, over several weeks, can recalibrate taste sensitivity, allowing individuals to accept lower-sodium foods without compromising perceived palatability [20].

Consumer awareness and literacy regarding sodium intake also play a critical role. Although general awareness of the association between salt intake and hypertension is relatively widespread, detailed knowledge remains limited. Many individuals are unable to identify recommended intake levels or recognize major sources of “hidden” sodium in everyday foods, such as bread, processed snacks, and ready meals. This discrepancy highlights a gap between perceived and actual exposure, which may reduce the effectiveness of behavior-based interventions in the absence of supportive environmental changes.

### 4.3. Composite Indices

To capture the multidimensional nature of salinogenic food environments, future research could benefit from approaches that integrate indicators across all environmental domains. In this context, the Salinogenic Environment Index (SEI) is proposed as a conceptual framework for the future quantification of the extent to which a given environment promotes excessive sodium exposure.

The proposed SEI would integrate indicators across the four domains of the framework: physical, economic, sociocultural, and policy/governance environments. Candidate indicators are presented in Table 1 and include, for example, sodium density in foods sold at retail and away from home, the availability and accessibility of low-sodium alternatives, population preferences regarding salt taste, sociocultural practices, and the strength of sodium-related policies. At this stage, the objective is to identify the dimensions and indicators that could support the future operationalization of salinogenic exposure.

In parallel, the policy/governance component could be assessed through a more specific policy implementation approach, informed by existing frameworks, such as the WHO SHAKE Technical Package [21]. This approach could consider dimensions such as sodium surveillance, food reformulation, nutrition labeling, public food procurement, consumer education, and the creation of supportive food environments. This policy-focused assessment would complement the broader SEI, characterizing the extent to which structural responses to salinogenic environments have been implemented.

It is important to emphasize that more empirical work is needed before the SEI or any related policy implementation measure can be used as a formal research or surveillance instrument. Future studies should establish the most appropriate indicator selection, measurement properties, scale, weighting, aggregation, reliability, validity, and comparability across different populations and food system contexts.

**Table 1:** Indicators and Measurement Approaches for the Salinogenic Food Environment.

Domain	Measurement Dimension	Indicator	Description	Data Sources/Methods	Relevance for Policy/Research
Physical	Food supply [15,22]	Sodium density in retail foods	Sodium content variability across similar products and categories	Food composition databases (e.g., EuroFIR), FoodSwitch, product label audits	Identifies reformulation potential and benchmarks industry performance
	Out-of-home exposure [12]	Menu sodium levels	Sodium content of meals in restaurants, takeaways, and catering settings	Menu audits, laboratory analysis, national surveys	Highlights high-risk food categories and supports regulation of OOH sector
	Institutional settings [23]	Compliance with sodium standards	Presence and adherence to sodium limits in schools, hospitals, and workplaces	Procurement policies, institutional audits	Enables controlled reduction of sodium exposure in public settings
	Population exposure [24]	24 h urinary sodium excretion	Objective biomarker of total sodium intake	24 h urine collection (gold standard)	Validates environmental exposure and tracks population-level intake
Economic	Affordability [25,26]	Price differentials	Relative cost of high-sodium vs. low-sodium foods	Retail price surveys, market data	Identifies economic barriers to healthy choices
	Market structure [15,16]	Availability of low-sodium alternatives	Market share and accessibility of reformulated or low-sodium products	Retail audits, sales data	Assesses effectiveness of reformulation policies
	Public procurement [23]	Nutrition criteria in purchasing	Inclusion of sodium thresholds in institutional procurement policies	Policy analysis, institutional guidelines	Leverages purchasing power to improve food environments
Sociocultural	Taste preferences [18]	Salt preference levels	Population-level preference for saltiness	Sensory testing, dietary surveys	Reflects adaptation to high-sodium environments
	Early-life exposure [27]	Childhood sodium exposure	Sodium content of foods consumed by children (e.g., school meals)	School meal assessments, dietary intake surveys	Indicates long-term habit formation risk
	Consumer awareness [28]	Sodium knowledge and perceptions	Awareness of intake recommendations and sources of sodium	Surveys, questionnaires	Identifies gaps between knowledge and behavior
Policy & Governance	Reformulation policies [29,30]	Voluntary vs. mandatory targets	Scope and enforcement of sodium reduction targets	Policy review, government reports	Assesses strength of regulatory frameworks
	Food labeling [31]	Front-of-pack labeling (FOPL)	Presence and type of interpretive labeling systems	Policy analysis	Influences consumer choice and industry reformulation
	Fiscal measures [32]	Taxes/subsidies	Implementation of fiscal policies targeting high-sodium foods	Government reports, economic evaluations	Evaluates impact on consumption patterns
	Monitoring systems [21]	Surveillance and evaluation	Existence of national sodium monitoring systems	WHO SHAKE framework, national reports	Supports accountability and policy effectiveness

## 5. Pathways: From Environment to Health

**Figure 2:**
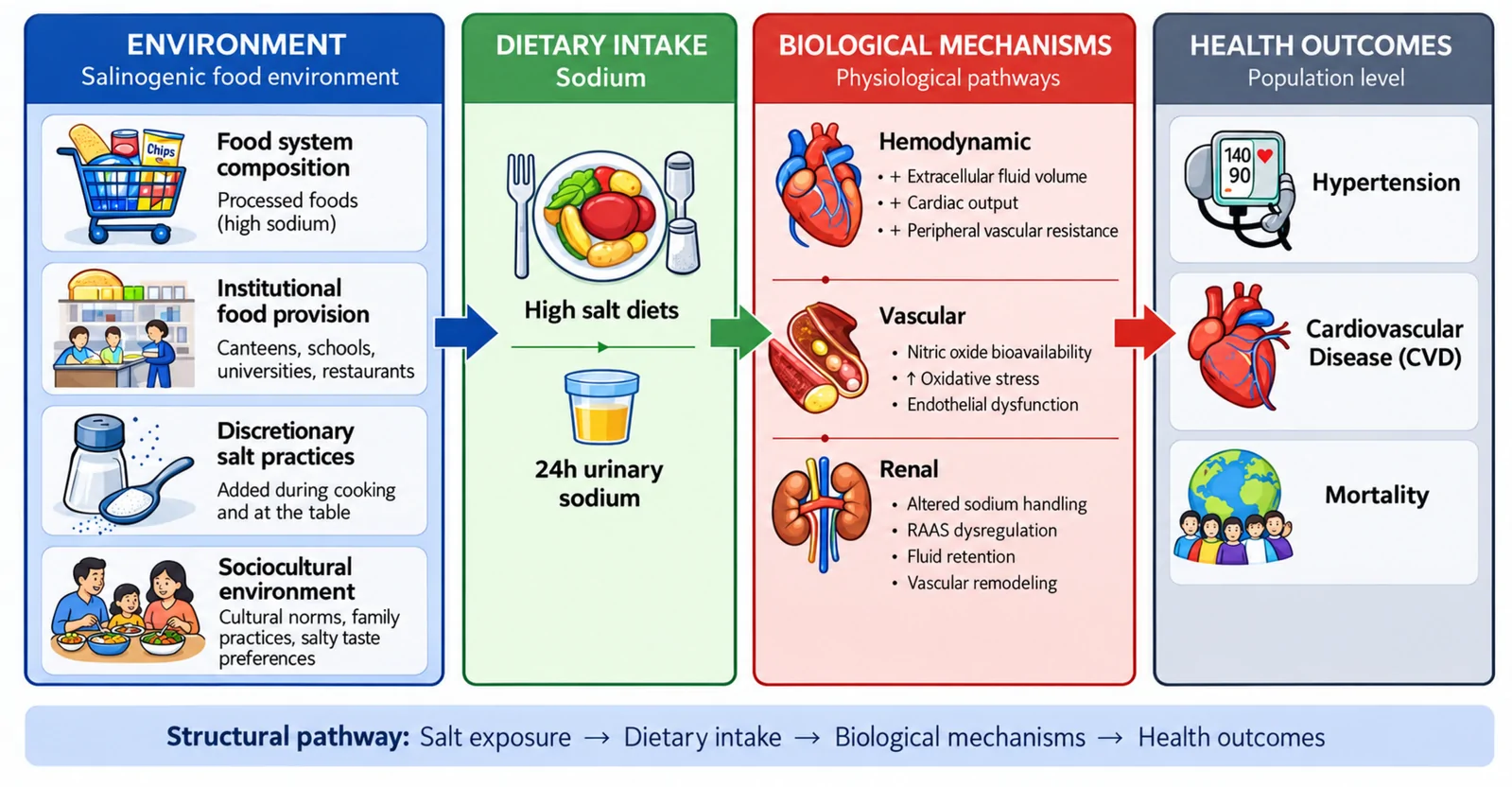
The salinogenic pathway linking food environments to cardiovascular health.

The health effects of salinogenic food environments can be conceptualized through a multilevel causal pathway that links environmental salt exposure to dietary intake, physiological responses, and population health outcomes (Figure 2). Building on the environmental domains described above, this pathway highlights how structurally embedded salt exposure translates into cardiovascular risk and other risks through interconnected behavioral and biological mechanisms [33]. This pathway emphasizes that dietary sodium intake is the principal mediator linking environmental exposure to downstream physiological and clinical outcomes.

Dietary salt intake, as the key mediator between environmental exposure and physiological response, is most accurately assessed through 24 h urinary sodium excretion, considered the gold standard method. This approach is based on the physiological principle that approximately 90 to 95% of ingested sodium is excreted in the urine over a 24 -h period under steady-state conditions [34]. Beyond 24 h urinary sodium excretion, sodium intake can also be estimated using alternative urinary and dietary assessment methods. Spot urine samples, including casual, morning, or timed samples, are less laborious and more feasible for large-scale studies, but require predictive equations, such as the Kawasaki or Tanaka formulas, to estimate 24 h sodium excretion. However, their accuracy is limited by hydration status, circadian variation, recent food intake, and interindividual differences in renal sodium metabolism, making them less reliable at the individual level [24,35]. Other urinary approaches, such as overnight urine samples, have also been used in salt reduction intervention studies [36]. Dietary methods, including 24 h recalls, food records, food diaries and food frequency questionnaires, are also used to estimate sodium intake and identify major dietary sources, although they may underestimate total intake because of under-reporting, incomplete food composition data and the difficulty of quantifying salt added during cooking or at the table [35,37].

A key behavioural mechanism linking environmental sodium exposure to dietary intake is behavioral adaptation through the development of salty taste preference. Sensitivity to and preference for salty taste emerge at approximately 4 months of age and appear to have an innate biological component. However, these early responses are progressively modified by prenatal influences and early dietary experiences. By around 2 years of age, children’s preference for salty foods exceeds that of adults, highlighting the interaction between biological predisposition and environmental exposure. These findings suggest that repeated exposure to high-sodium foods during early life may reinforce preferences for salty tastes and contribute to long-term dietary patterns [27]. In this context, a key intermediate step linking environmental sodium exposure to dietary intake is behavioral adaptation, particularly through the development of salt taste preference. Repeated exposure to high levels of salt, whether through diet or potentially through salinogenic environments promotes sensory habituation and increases preference for salty tastes, reinforcing dietary patterns over time. This process may create a feedback loop in which higher intake further strengthens preference [18,20].

This behavioral adaptation is biologically grounded in the mechanisms of salt taste perception, which involve direct ionic detection, taste signal transduction and cognitive integration. According to Yang et al., sodium ions can activate taste receptor cells mainly through epithelial sodium channels, particularly the amiloride-sensitive epithelial sodium channel pathway, which allows sodium influx, cell depolarization and transmission of salty taste signals. In parallel, amiloride-insensitive mechanisms may also contribute to salt perception, involving other ion channels and receptor-mediated pathways that respond to sodium and other mineral ions. The authors further emphasize that perceived saltiness is not determined only by sodium concentration but can be modulated by interactions with other taste qualities, especially umami and sourness, as well as by olfactory, tactile, visual, auditory and trigeminal cues. Therefore, salty taste perception results from the integration of peripheral taste transduction mechanisms with multisensory and cognitive processes, which provides the scientific basis for salt-reduction strategies that enhance saltiness perception without necessarily increasing sodium content [38].

Excessive sodium intake influences cardiovascular health through a network of interrelated physiological mechanisms encompassing hemodynamic, vascular, and renal pathways. At the systemic level, high sodium intake increases extracellular fluid volume, resulting in elevated cardiac output and, over time, increased peripheral vascular resistance, thereby contributing to sustained elevations in blood pressure—an effect that is particularly pronounced in salt-sensitive individuals [39]. Salt sensitivity is more prevalent among specific populations, including older adults, individuals of African ancestry, and patients with chronic kidney disease, obesity, diabetes, or metabolic syndrome. These groups exhibit alterations in renal sodium handling, impaired pressure–natriuresis relationships, and enhanced neurohormonal activation, which collectively contribute to greater blood pressure elevations in response to sodium intake. Genetic predisposition and low dietary potassium intake may further modulate this susceptibility. Importantly, salt sensitivity has been recognized as an independent cardiovascular risk factor, even in normotensive individuals, underscoring its relevance beyond conventional blood pressure classification [40,41,42].

Beyond its effects on blood pressure, sodium also exerts direct effects on vascular function by reducing endothelial nitric oxide bioavailability and increasing oxidative stress, thereby promoting endothelial dysfunction—an early hallmark of atherosclerosis and a predictor of cardiovascular events [39,43]. Renal mechanisms further reinforce these effects, as chronic high sodium intake alters sodium handling and disrupts the regulation of the renin–angiotensin–aldosterone system, particularly in susceptible populations. These alterations contribute to fluid retention, vascular remodeling, and progressive renal impairment, thereby amplifying cardiovascular risk. Collectively, these processes lead to increased arterial stiffness, cardiac remodeling, and elevated cardiovascular risk [39].

At the population level, these mechanisms translate into a substantial burden of hypertension and cardiovascular disease (CVD). CVDs remain the leading cause of mortality globally, accounting for approximately 19.8 million deaths in 2022 (32% of all deaths) [44]. Hypertension is a major underlying driver, affecting an estimated 1.4 billion adults worldwide in 2024, with prevalence nearly doubling since 1990 [45].

In this context, the salinogenic food environment provides a critical explanatory framework for understanding how these outcomes are structurally produced. By systematically promoting high sodium exposure across multiple settings, these environments can contribute to sustained increases in blood pressure and, consequently, to higher incidence of CVD and mortality. This underscores the need to move beyond individual-level approaches and address the environmental determinants that underpin excessive sodium consumption. Accordingly, the salinogenic food environment framework shifts prevention strategies from treating sodium intake as an individual behavior to addressing it as a structural property of contemporary food systems.

## 6. Research at the Level of the Physical Food Environment

The physical food environment is a central driver of salt exposure because it determines which foods are available, affordable, and routinely consumed in both retail and out-of-home settings [46,47].

Dietary sodium intake includes sodium naturally present in foods, but also, and more importantly, sodium added as salt during culinary preparation, at the table, and during industrial food processing. However, the relative contribution of these sources varies across countries and according to the level of economic development, reflecting differences in food systems, culinary practices, dietary patterns, and reliance on processed foods. In their systematic review of 80 studies across 34 countries, Bhat et al. found marked international variation in the contribution of discretionary salt versus non-discretionary sodium from the food supply. More than half of daily salt intake came from discretionary sources in countries such as Brazil, China, Costa Rica, Guatemala, India, Japan, Mozambique, Romania, and Benin. In countries such as Portugal, Jordan, Taiwan, South Korea, and Turkey, discretionary salt use contributes between 25% and 50% of total sodium intake. The same review also identified a strong inverse association between national income and discretionary salt reliance: for every US$10,000 increase in GDP per capita, the proportion of salt obtained from discretionary sources fell by 8.7 percentage points. Across many higher-income settings, the leading food contributors were bread products, cereal and grain foods, meat products, and dairy products, underscoring how salt exposure is built into the physical food environment rather than arising only from individual behavior [48].

In high-income regions such as Europe, sodium intake is predominantly derived from industrial food systems. Evidence indicates that 70–75% of total sodium intake originates from processed and commercially prepared foods, including ultra-processed products and out-of-home meals [49]. Within this context, ultra-processed foods represent a major contributor to sodium exposure.

Empirical data from European populations reinforce this pattern. In Spain, ultra-processed foods account for 47.8% of total sodium intake in children, with processed foods contributing an additional 35.5%, resulting in more than 80% of sodium intake originating from industrially processed products. Within this sample, UPF contributed most strongly to sodium from ready-to-eat dishes, sugars and pastries, vegetables, appetizers, sauces, and condiments, in some of these groups accounting for 56.5% to 100% of sodium intake. White bread, Serrano ham, cured sausages, and milk were among the top individual contributors [50].

Similar patterns are observed across Europe, where staple products such as bread contribute disproportionately to sodium intake, accounting for up to 20–40% of total intake in several countries [48].

Evidence from the United States further corroborates the central role of the retail and prepared food supply. FDA consumer information indicates that over 70% of dietary sodium comes from packaged and prepared foods. A recent analysis based on the NHANES (National Health and Nutrition Examination Study) estimated that store-bought foods contributed 62%, restaurant-prepared foods 26%, and other foods 12% of sodium in American diets. The same study identified sandwiches, tortilla-based products, pizza, poultry, soups, and breads as the main contributors [51,52].

Brazilian data show that sodium largely derives from table salt, while the contribution from processed and ultra-processed products is increasing. Nilson et al. reported that the average sodium availability in Brazilian households increased from 3.9 g to 4.7 g per 2000 kcal/day between 2002–2003 and 2017–2018, remaining more than double the recommended intake. In 2017–2018, processed culinary ingredients contributed 55% of total available sodium, while processed foods contributed 13.6% and ultra-processed foods 20.3%; this latter percentage increased over time, from 16.1% in 2002–2003 to 20.3% in 2017–2018. These findings are important for analyses of salinogenic environments, as they show that modernization of the food system does not simply replace salt consumption, instead, many populations experience layered exposure, in which discretionary salt consumption remains high while processed foods become increasingly important [53].

Portuguese data show that 29.2% of sodium intake comes from salt added during cooking, while key components of the physical food environment—such as bread (18.0%), soup (8.2%), and processed meat (7.0%)—collectively account for a significant proportion of intake [54].

Retail-level evidence further supports the salinogenic nature of the Portuguese food environment. An analysis of more than 4000 pre-packaged food products available in Portugal found consistently high salt levels across all categories, with particularly elevated values in processed meats (~2.20 g/100 g), sauces (~1.70 g/100 g), and ready-to-eat meals (~1.10 g/100 g), indicating that a large proportion of foods available to consumers contain sodium levels above recommended thresholds [55]. More recent data further confirm this pattern, showing that 62.7% of packaged food products exceed WHO sodium benchmarks, highlighting the widespread availability of high-sodium products across the retail environment [22].

A recent population study among older adults found that individuals with higher consumption of ultra-processed foods were more likely to add salt at the table, indicating that exposure to high-sodium food environments can alter taste preferences and reinforce salt-seeking behaviors [56]. This suggests that the physical food environment not only directly increases sodium intake through food composition but can also indirectly amplifies consumption by shaping individual preferences and behaviors over time.

The out-of-home sector is consistently identified as a high-sodium environment. In China, a large cross-sectional survey of 8131 restaurant dishes from 192 restaurants found a median sodium content of 487.3 mg/100 g and 2543.7 mg per serving, with 62.6% exceeding the recommended daily intake of 2000 mg. Average sodium per serving was 3331.2 mg, equivalent to 222% of the daily adequate intake. Only 45.8% of dish sodium came from cooking salt itself, major additional contributions from monosodium glutamate (17.5%), food ingredients (17.1%), soy sauce (9.4%), and other condiments or seasonings (10.2%), illustrating that restaurant sodium exposure often derives from multiple layered salt sources rather than visible salt alone [57].

A review of United States restaurant food monitoring methods reported that average daily sodium intake was 3623 mg/day among individuals consuming at least one restaurant meal, compared with 2999 mg/day among those who did not [58]. Additional analysis report sodium values in main dishes averaging around 1512 mg in major United State restaurant chains [59], and fast-food meal purchases averaging approximately 1292 mg of sodium in adults and 1128 mg in adolescents [60]. These findings indicate that restaurant meals alone can account for a substantial proportion of the recommended daily sodium intake, even before considering total daily consumption.

Institutional food service environments, including workplace canteens, university cafeterias, and school canteens, represent important components of the physical food environment. Available evidence suggests that these settings can act as significant sources of sodium exposure while also offering opportunities for structural intervention. For example, studies conducted in workplace canteens have reported salt contents of approximately 2.8 to 3.8 g per meal, representing a significant proportion of the recommended daily intake [61]. Similarly, assessments of school meals demonstrate equally high salt levels in prepared meals [62,63].

School meals are particularly important because they influence sodium exposure early in life and can normalize salty taste preferences. A Portuguese study analyzing 798 school meal samples found high salt levels, particularly in bread (1.35 g/100 g), followed by soups (0.72–0.80 g/100 g) and main courses (0.71–0.97 g/100 g). Overall, total meals contained between 2.83 and 3.82 g of salt. Despite this, many students did not perceive the meals as overly salty, suggesting adaptation to high-salt food environments [64].

In Portuguese university canteens, meals consisting of soup and a main course contained, on average, 2.8 g of salt per serving, corresponding to approximately 53% of the WHO maximum daily sodium recommendation. Salt addition was often performed “by instinct,” highlighting the role of food preparation practices and lack of standardization in institutional settings [65].

Intervention studies demonstrate that this source of exposure is modifiable. In a Portuguese university canteen trial, the implementation of a salt-dosing device resulted in reductions in added salt exceeding 30%, specifically −34.3% in soup, −41.1% in fish dishes, and −48.0% in meat dishes, without negatively affecting consumer satisfaction or increasing food waste [63]. These findings illustrate that structural and operational changes in food preparation can substantially reduce sodium exposure while maintaining acceptability.

Snacks are another important component of the physical food environment, as they are widely available, aggressively marketed, and frequently consumed outside structured meals. At the total diet level, recent US estimates identify salty snacks as accounting for approximately 4.1% of total sodium intake, placing them among the leading contributing categories [66].

Taken together, the literature shows that the physical food environment promotes high sodium intake through multiple, overlapping pathways. Salt exposure should not be understood solely as a matter of individual preference or knowledge. Instead, the evidence supports a structural interpretation in which modern food environments function as salinogenic systems, normalizing the availability and consumption of high-sodium products across retail, food service, and institutional contexts, while simultaneously shaping taste preferences and reinforcing salt-seeking behaviors over time.

## 7. Research at the Level of the Economic Environment

The economic environment is a core determinant of salinogenic food environments, as it shapes which foods are produced, priced, promoted and ultimately consumed. Sodium intake is therefore not solely the result of individual preferences or knowledge but is strongly influenced by structural economic conditions that make salt-rich foods cheaper, more accessible, and more commercially attractive than healthier alternatives. These dynamics operate through interconnected mechanisms, including food pricing and affordability, industry-level economic incentives, the concentration of economic constraints among disadvantaged populations, and the presence (or absence) of regulatory and fiscal policies capable of correcting these imbalances [67,68].

Food pricing is one of the most important determinants of dietary behavior. In high-income countries, there is evidence that healthier dietary patterns are more expensive than less healthy alternatives. A systematic review and meta-analysis by Mayuree Rao et al. found that healthier diets cost approximately US$1.48 more per person per day compared to less healthy diets [25]. Furthermore, foods with high energy density tend to be the least expensive per unit of energy, favoring lower-quality diets among populations with budget constraints [69]. This is particularly relevant for sodium exposure, as ultra-processed foods, often high in salt, are among the cheapest and most shelf-stable options available in the food system.

The widespread use of salt in the food industry is strongly driven by economic incentives. Salt is a low-cost ingredient with multiple technological functions, including enhancing palatability, improving texture, and extending shelf life, making it highly attractive for food manufacturing. As a result, high-salt processed foods are not only a nutritional concern but also a commercially efficient product form. This is reflected in the persistent availability of sodium-rich products despite sustained policy attention [5].

Evidence on price elasticity further reinforces the role of economic factors in shaping dietary choices. A systematic review based on 160 studies found that food demand responds significantly to price changes, a 10% increase in the price of unhealthy foods can reduce consumption by 6 to 10% [70].

In the European context, these dynamics have been amplified by recent economic pressures. Following the 2022–2024 period of food inflation, Eurostat reported that annual inflation in the European Union increased from 0.7% in 2020 to 9.2% in 2022, before declining to 6.4% in 2023 and 2.6% in 2024. Rising prices of essential goods disproportionately affected lower-income populations. The European Commission further noted that, although food inflation stabilized in 2024, overall price levels remained elevated, and the modest reductions were not perceived by consumers, particularly among lower-income groups, thereby limiting access to healthier and more sustainable food options [71].

Socioeconomic inequalities are a consistent determinant of sodium intake. A systematic review and meta-analysis reported that individuals of lower socioeconomic status consume, on average, 14% more sodium than those of higher socioeconomic status. Additionally, approximately 67% of studies report higher sodium intake among disadvantaged populations [72].

These disparities are driven by structural economic barriers, including: (a) affordability constraints, as healthier foods generally require greater financial investment; (b) availability constraints, with low-income areas more frequently exposed to environments dominated by processed foods; (c) time constraints, as lower-income households face higher opportunity costs, favoring convenience foods; and (d) market exposure, including aggressive marketing and the availability of low-cost processed foods. Together, these factors increase reliance on inexpensive, ultra-processed, and high-salt foods, reinforcing dietary inequalities [26,72,73].

Economic and regulatory policies are essential tools for reducing population salt intake. The World Health Organization identifies sodium reduction as one of the most cost-effective interventions for preventing cardiovascular diseases [74]. Fiscal instruments represent an additional policy lever to modify both consumption patterns and industry behavior. These include taxes on high-salt foods, levies based on sodium content, and targeted taxation of product categories such as snacks, condiments, and ultra-processed foods.

Hungary provides one of the most cited examples, with the introduction of the Public Health Product Tax in 2011. This policy applies to products high in nutrients of concern, including sodium, and has been associated with reductions in the consumption of taxed products as well as reformulation by manufacturers [19].

A review on the feasibility and effectiveness of taxing salt and high-salt foods states that modeling studies demonstrate potential benefits, and that taxes based on the salt content of all foods may have a greater impact than taxes applied only to specific categories. However, evidence in a real-world context is limited with possible unintended effects such as substitution with cheaper high-salt products [32]. This concern is supported by a recent modelling study from Iran using household expenditure data, which found that taxing salty foods was ineffective in reducing total salt intake. After a simulated 20% tax, total salt intake was projected to increase by approximately 125 g per month, as households shifted toward lower-quality and cheaper salty foods. The authors concluded that, although such taxes may increase government revenue, they may fail to reduce salt consumption unless carefully designed to account for substitution effects and cross-price elasticities [75].

In addition to fiscal measures, such as taxes and subsidies, strategies including food reformulation and public procurement policies can reduce salt consumption and act as key levers to reshape salinogenic environments.

National salt reduction programmes have demonstrated measurable success. The United Kingdom salt reduction initiative, for example, achieved an approximate 15% reduction in population salt intake, accompanied by declines in blood pressure and cardiovascular mortality [76].

Public procurement policies, particularly in schools, hospitals, and other institutional settings, represent an additional economic lever. These interventions can influence both food supply and consumption patterns at scale, while also creating market incentives for product reformulation as suppliers adapt to meet procurement standards [77]. Although economic evaluations of public food procurement policies remain relatively limited, available modelling studies suggest that these measures may generate health and economic benefits. For example, an analysis conducted in Los Angeles County estimated that sodium-reduction procurement standards in government-operated or government-funded food service settings could reduce mean sodium intake, improve blood pressure control and generate annual savings in direct healthcare costs [78].

Overall, the economic environment can be considered salinogenic when market structures systematically favor the affordability, availability, and profitability of sodium-rich foods over healthier alternatives.

## 8. Research at the Level of the Sociocultural Environment

The sociocultural environment constitutes a central domain of salinogenic environments, as sodium intake is not determined solely by individual choice but is shaped by learned preferences, family practices, culinary traditions, social norms, socioeconomic position, and the symbolic meaning of food [73,79,80].

Salt is deeply embedded in food cultures worldwide. Historically, it played a fundamental role in food preservation through salting, curing, and fermentation, practices that remain integral to many traditional cuisines [81]. Consequently, many sodium-rich foods are not perceived merely as unhealthy products, but rather as traditional, familiar, festive, or identity-defining components of cultural heritage, making reductions in salt intake particularly challenging despite widespread awareness of its health risks [28,79].

Within salinogenic environments, family eating practices represent one of the earliest mechanisms through which sodium exposure becomes normalized. Children are exposed to the sodium profile of the household diet through repeated exposure to meals prepared according to family traditions. Evidence from early food learning demonstrates that repeated exposure increases food acceptance, and this principle is directly applicable to salty foods, whereby the repeated use of salt to enhance palatability reinforces preference for higher sodium levels over time [82,83]. Through these early dietary experiences, sociocultural influences are transmitted across generations, contributing to the persistence of salinogenic environments.

Cultural traditions and food practices vary substantially across regions, resulting in distinct patterns of sodium consumption. In East Asian countries, sodium intake is strongly linked to traditional culinary practices and the widespread use of salt-based seasonings. In China, the majority of sodium intake derives from salt added during home cooking, with average intake remaining high, at approximately 9.3 g/day, reflecting the central role of discretionary salt in traditional cuisine [84]. Similarly, in Japan, dietary patterns are characterized by a high sodium intake from soy sauce, miso, pickled vegetables, and salted fish [85]. Total salt intake remains elevated (approximately 9–11 g/day), with a significant proportion derived from soy-based condiments, accounting for up to 66% of total sodium intake in some analyses. These sources are deeply embedded in Japanese culinary identity and in the structure of daily meals [86].

In Portugal, the national cuisine includes several traditional or commonly consumed foods with a high salt content, such as salted cod, cured cheeses, olives, breads, soups, sausages, cured meats, ham, and salted or cured fish and meat preparations. Bread is a significant source of salt, although national legislation limits salt content in bread to 1.4 g per 100 g, exemptions for imported and traditional products highlight the tension between public health policies and the preservation of culinary traditions [14,87].

In Brazil, traditional dietary habits also play an important role in shaping sodium intake patterns. Evidence suggests that discretionary salt use remains a major contributor to sodium consumption, particularly through salt added during food preparation and at the table [56].

Migration studies further illustrate the persistence of cultural dietary patterns. Qualitative research among Nigerian adults living in the United States showed that sodium sources differed from the dominant American pattern, as participants maintained traditional eating habits and relied less on processed foods [88]. These findings illustrate that cultural food practices may persist despite changes in the surrounding food environment, highlighting the durability of sociocultural influences on sodium exposure.

Within the salinogenic environment framework, these sociocultural factors act as upstream drivers that normalize high salt exposure and reinforce long-term dietary patterns.

## 9. Research at the Level of the Policy & Governance Environment

The policy and governance environment is a central determinant of salinogenic food environments, as it defines the regulatory frameworks, incentives, and institutional responsibilities that shape the food supply. The most effective strategies are comprehensive and combine product reformulation, nutrition standards for public food procurement, interpretive nutrition labeling, and fiscal instruments [1,21].

Dietary reformulation represents one of the most important structural responses to excessive sodium intake, particularly given that a large proportion of dietary sodium originates from processed foods. By progressively reducing sodium content across food categories, reformulation strategies can decrease population exposure independently of individual behavior [21].

Modelling studies suggest that more ambitious reformulation strategies could yield substantial health gains. For instance, sodium reduction interventions in Australia were estimated to generate significant gains in quality-adjusted life years (QALYs), alongside considerable healthcare cost savings [89]. A systematic review estimated that mandatory reformulation could reduce salt intake by approximately 1.45 g/day, compared with about 0.8 g/day for voluntary reformulation. More recent evidence continues to support the conclusion that mandatory or strongly monitored policies have a greater impact than isolated voluntary commitments [29,30].

In Portugal, national legislation has established a maximum limit of 1.4 g of salt per 100 g of bread. A modelling study by Goiana-da-Silva et al., estimated that reducing this limit to 1.0 g/100 g and applying it to all bread products—including imported and traditional varieties—could decrease salt consumption by approximately 3.6 tonnes per day and prevent around 286 deaths annually [87]. This example illustrates how relatively small mandatory changes in widely consumed, low-cost staple foods can produce measurable population-level health benefits, without relying on individual behavior change or high levels of health literacy.

The United Kingdom is frequently cited as a successful example of a national sodium reduction strategy. Its program combined voluntary category-specific targets, industry engagement, public awareness campaigns, and systematic monitoring through 24-h urinary sodium assessments. This multifaceted approach resulted in a reduction in average salt intake from 9.5 to 8.1 g/day, with gradual product reformulation identified as a key driver of change [76].

Evidence from other regions supports the scalability of reformulation policies. In Brazil, a voluntary national program targeting processed foods achieved reductions of 8–34% in sodium content across multiple food categories between 2011 and 2017 [90]. Similarly, coordinated regional initiatives in Latin America have demonstrated high levels of industry compliance, with up to 86% of products meeting established sodium targets in some countries [91]. These findings indicate that substantial reductions in sodium content are both technically feasible and achievable at scale through coordinated policy action.

To support these efforts, the World Health Organization has established global sodium benchmarks across multiple food categories, updated in 2024 to include 70 subcategories. These benchmarks provide a technical foundation for national target-setting and facilitate policy implementation and industry accountability [92]. Within this framework, reformulation should be understood as a governance intervention that directly modifies the structural composition of the food environment.

Public food procurement policies constitute another critical mechanism for shaping food environments at scale. Governments and public institutions provide meals to large population groups, including schoolchildren, hospital patients, and other institutionalized populations. The integration of mandatory nutritional criteria, including sodium limits, into procurement contracts has demonstrated potential to reduce dietary sodium exposure while simultaneously creating demand for reformulated products [23]. Notably, more than 70% of countries with national salt reduction strategies have implemented interventions in institutional settings such as schools and hospitals [17].

Beyond their direct impact on dietary intake, procurement policies generate important indirect effects. These include incentivizing industry reformulation, increasing the availability of healthier food options, and contributing to the normalization of nutritional standards. However, their effectiveness depends on robust governance structures, including monitoring systems, enforcement mechanisms, and institutional capacity [1,93].

Nutritional labeling, particularly interpretive front-of-pack labeling, plays a key role in addressing information asymmetries between industry and consumers. Labeling schemes such as traffic light systems, Nutri-Score, and warning labels (e.g., “high in sodium”) enhance the visibility of sodium content in processed foods and improve consumer understanding. Evidence indicates that the implementation of interpretive labels is associated not only with improved consumer choices but also with product reformulation, as manufacturers seek to avoid negative classifications [31,94].

A meta-analysis has demonstrated that front-of-pack labeling is associated with improved dietary choices and reduced purchasing of products with unfavorable nutritional profiles [95]. In salinogenic environments, where sodium is often embedded in foods not perceived as salty, interpretive labeling transforms sodium content into a visible and comparable characteristic, thereby enabling both consumer decision-making and regulatory pressure on the food industry.

Overall, the policy and governance environment represents the structural level of prevention within salinogenic food systems. Reformulation programs alter the composition of the food supply; procurement standards reshape institutional food environments; labeling systems reduce information asymmetry and increase transparency; and fiscal policies modify economic incentives. When implemented in an integrated manner, these strategies have the potential to shift responsibility from individual behavior toward systemic, public health-oriented food governance, thereby addressing the structural drivers of salinogenic environments.

## 10. Conclusions

The concept of the salinogenic food environment proposed in this paper provides a novel ecological framework for understanding excessive sodium consumption as a structurally determined phenomenon rather than solely the result of individual choice. Building on established environmental health models, particularly the ANGELO framework, this conceptualization highlights how physical, economic, sociocultural, and policy environments interact to promote chronic overexposure to dietary sodium across populations.

The evidence reviewed demonstrates that contemporary food systems systematically favor high sodium intake through the widespread availability of processed and ultra-processed foods, the high salt content of out-of-home meals, economic incentives that support the use of salt in food production, and sociocultural norms that normalize salty taste preferences from early life onwards. These environmental drivers contribute not only to elevated sodium intake but also to the development and reinforcement of salty taste preferences, thereby sustaining cardiovascular risk through interconnected behavioral, physiological, and structural pathways.

Importantly, this framework reinforces the need to shift public health approaches away from strategies focused predominantly on individual responsibility and nutrition education. Although consumer awareness remains relevant, the evidence suggests that educational interventions alone are insufficient in environments where high-sodium foods are affordable, accessible, culturally normalized, and institutionally reinforced. Instead, reducing population salt intake requires comprehensive structural interventions capable of modifying the food environment itself.

The salinogenic food environment framework also offers a practical basis for research, surveillance, and policy development. The proposed multidimensional indicators and composite measures, such as the Salinogenic Environment Index, may support the monitoring and comparison of sodium exposure across settings and populations, while facilitating the evaluation of policy effectiveness. At the governance level, the evidence consistently supports integrated structural policies, including food reformulation, public procurement standards, interpretive front-of-pack labeling, fiscal measures, and robust sodium surveillance systems based on 24-h urinary sodium excretion.

By explicitly framing sodium exposure as an environmental and systemic phenomenon, the salinogenic food environment complements existing food environments frameworks and provides a useful conceptual basis for research, policy development, and public health action. Analogous to the obesogenic environment paradigm in obesity research, this framework may help advance the understanding, measurement, and prevention of excessive sodium consumption.

The conceptual and narrative nature of this review should be considered when interpreting the proposed framework. The literature-selection process was guided by conceptual relevance and therefore may be subject to selection bias. In addition, the proposed environmental domains and measurement indicators remain conceptual and require empirical validation across populations and food-system contexts.

Future research should focus on validating operational measures of salinogenic exposure, including the development of composite indices applicable across diverse sociocultural and economic contexts. Longitudinal and multilevel studies are also needed to better understand how environmental sodium exposure interacts with taste adaptation, socioeconomic inequalities, and cardiovascular risk across the life course. Ultimately, recognizing and addressing the salinogenic nature of contemporary food systems shift excessive sodium consumption from being viewed primarily as an individual behavior to being understood as a structural property of the environments in which food choices are made. This perspective provides a stronger foundation for developing effective and sustainable strategies to reduce population sodium intake and the global burden of cardiovascular disease.

## Data Availability

No new data were created or analyzed in this study. Data sharing is not applicable to this article.
